# Prediction of Bladder Outcomes after Traumatic Spinal Cord Injury: A Longitudinal Cohort Study

**DOI:** 10.1371/journal.pmed.1002041

**Published:** 2016-06-21

**Authors:** Chiara Pavese, Marc P. Schneider, Martin Schubert, Armin Curt, Giorgio Scivoletto, Enrico Finazzi-Agrò, Ulrich Mehnert, Doris Maier, Rainer Abel, Frank Röhrich, Norbert Weidner, Rüdiger Rupp, Alfons G. Kessels, Lucas M. Bachmann, Thomas M. Kessler

**Affiliations:** 1 Neurology, Spinal Cord Injury Center, University of Zürich, Balgrist University Hospital, Zürich, Switzerland; 2 Specialization School in Physical Medicine and Rehabilitation, Department of Clinical-Surgical Sciences, University of Pavia, Rehabilitation Unit, Fondazione IRCCS Policlinico San Matteo, Pavia, Italy; 3 Neuro-Urology, Spinal Cord Injury Center, University of Zürich, Balgrist University Hospital, Zürich, Switzerland; 4 Brain Research Institute, University of Zürich, Zürich, Switzerland; 5 Spinal Cord Unit, IRCCS Fondazione Santa Lucia, Rome, Italy; 6 Department of Experimental Medicine and Surgery, Tor Vergata University, Rome, Unit for Functional Urology, Department of Urology, Policlinico Tor Vergata, Rome, Italy; 7 Spinal Cord Injury Center, BG-Trauma Center, Murnau, Germany; 8 Spinal Cord Injury Center, Hohe Warte, Bayreuth, Germany; 9 Berufsgenossenschaftliche Kliniken Bergmannstrost, Zentrum für Rückenmarkverletzte und Klinik für Orthopädie, Halle, Germany; 10 Spinal Cord Injury Center, Heidelberg University Hospital, Heidelberg, Germany; 11 Department of Anesthesiology and Pain Medicine, Maastricht University Medical Center, Maastricht, the Netherlands; 12 Medignition Inc., Research Consultants, Zürich, Switzerland; Queen Mary University of London, UNITED KINGDOM

## Abstract

**Background:**

Neurogenic bladder dysfunction represents one of the most common and devastating sequelae of traumatic spinal cord injury (SCI). As early prediction of bladder outcomes is essential to counsel patients and to plan neurourological management, we aimed to develop and validate a model to predict urinary continence and complete bladder emptying 1 y after traumatic SCI.

**Methods and Findings:**

Using multivariate logistic regression analysis from the data of 1,250 patients with traumatic SCI included in the European Multicenter Spinal Cord Injury study, we developed two prediction models of urinary continence and complete bladder emptying 1 y after traumatic SCI and performed an external validation in 111 patients. As predictors, we evaluated age, gender, and all variables of the International Standards for Neurological Classification of Spinal Cord Injury (ISNCSCI) and of the Spinal Cord Independence Measure (SCIM). Urinary continence and complete bladder emptying 1 y after SCI were assessed through item 6 of SCIM. The full model relies on lower extremity motor score (LEMS), light-touch sensation in the S3 dermatome of ISNCSI, and SCIM subscale respiration and sphincter management: the area under the receiver operating characteristics curve (aROC) was 0.936 (95% confidence interval [CI]: 0.922–0.951). The simplified model is based on LEMS only: the aROC was 0.912 (95% CI: 0.895–0.930). External validation of the full and simplified models confirmed the excellent predictive power: the aROCs were 0.965 (95% CI: 0.934–0.996) and 0.972 (95% CI 0.943–0.999), respectively. This study is limited by the substantial number of patients with a missing 1-y outcome and by differences between derivation and validation cohort.

**Conclusions:**

Our study provides two simple and reliable models to predict urinary continence and complete bladder emptying 1 y after traumatic SCI. Early prediction of bladder function might optimize counselling and patient-tailored rehabilitative interventions and improve patient stratification in future clinical trials.

## Abstract 2

Thomas Kessler and colleagues identify and validate two models to predict urinary continence and complete bladder emptying one year after traumatic spinal cord injury.

## Author Summary

**Why Was This Study Done?:**

- Neurogenic bladder dysfunction is one of the most common and devastating sequelae of traumatic spinal cord injury.
- Recovery of bladder function represents an absolute priority for individuals affected by spinal cord injury, and it is often considered to be more important than recovery of ambulation.
- There is a valid rule to predict locomotion and upper limb function in patients with spinal cord injury, but no similar tool for bladder function is available.

**What Did the Researchers Do and Find?:**

- Based on data from 1,250 patients with traumatic spinal cord injury included in the European Multicenter Spinal Cord Injury (www.emsci.org) study, we derived two simple and reliable models to predict urinary continence and complete bladder emptying 1 y after traumatic spinal cord injury.
- Our models have been validated in an independent cohort of 111 patients with traumatic spinal cord injury.

**What Do These Findings Mean?:**

- Our models allow early prediction of bladder outcome after traumatic spinal cord injury, which is crucial to counsel patients, to set rehabilitative goals, and to orient patient-tailored interventions.
- Our models could help promptly identifying patients who are not likely to have favourable bladder outcomes despite standard therapies and who could benefit more from investigational therapies.
- Our models may improve patient stratification in future clinical trials assessing treatments for bladder dysfunction in patients with traumatic spinal cord injury.

## Introduction

Traumatic spinal cord injury (SCI) affects each year 15–53 new individuals per million in Western countries and often results in severe lifelong disability and considerable burden on the health care system [1–4]. Most patients with SCI develop neurogenic bladder dysfunction, which represents one of the most devastating sequelae for patients’ quality of life [1,5] and might lead to complications such as recurrent urinary tract infections (UTIs), urethral strictures, calculus disease, hydronephrosis, and renal failure. In the past, systemic complications deriving from urinary tract dysfunction were accountable for more than 40% of deaths among individuals affected by SCI [6]. The introduction of intermittent self-catheterization combined with antimuscarinic treatment and the use of regular urodynamic investigation has since revolutionized the care of patients with SCI, reducing the mortality due to urinary tract diseases to about 13% [6,7]. It follows that early diagnosis and treatment of neurogenic bladder dysfunction is essential to prevent irreversible deterioration of urinary tract function and potential life-threatening complications [8,9]. However, little is known about bladder function in the acute phase of SCI, and the urological assessments are often postponed to a chronic stage, i.e., several months post injury [10].

Neurourological management aims to preserve or improve upper urinary tract function, control UTIs, and maintain a low-pressure bladder that is both continent and capable of emptying completely [8,9,11]. The recommended assessment according to the European Association of Urology (EAU) Guidelines on Neuro-Urology [8] includes history taking, physical examination, bladder diary, urinalysis and urine culture, blood chemistry, uroflowmetry, ultrasonography (postvoid residual and upper urinary tract morphology), videourodynamic investigation (assessment of detrusor and bladder outlet function, compliance, and vesicoureterorenal reflux), urethrocystoscopy, and bladder washing cytology [8,9].

Recovery of bladder function represents an absolute priority for individuals affected by SCI, and it is often considered to be more important than recovery of walking or reduction of chronic pain [5]. Early definition of bladder function prognosis is essential to counsel patients, to set rehabilitative goals, and to orient a patient-tailored intervention [12,13]. Moreover, prediction of bladder function might improve the stratification of patients for future clinical trials [14]. However, a reliable urological prognosis is actually impossible, since appropriate predictive factors have not been identified and no predictive algorithm is available.

We hypothesized that bladder function after traumatic SCI is predictable, similarly to other clinical outcomes such as locomotion [15] and upper limb function [16]. Therefore, we employed a large dataset of patients with SCI from a European prospective observational multicentre study to develop two prediction models for urinary continence and complete bladder emptying 1 y after traumatic SCI. At a later stage, we performed an external validation of our models in an independent clinical dataset.

## Methods

### Study Design and Patient Population

**Fig 1:**
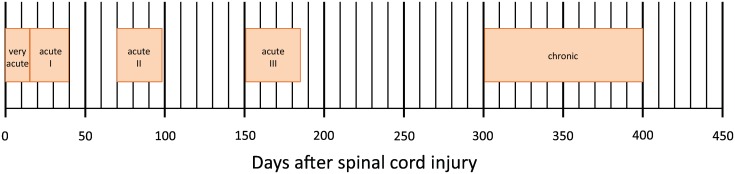
Time schedule of assessments in days after spinal cord injury of the European Multicenter Study about Spinal Cord Injury (EMSCI, www.emsci.org).

Data were derived from the European Multicenter Study about Spinal Cord Injury (EMSCI) database (www.emsci.org) (ClinicalTrials.gov Identifier: NCT01571531). Started in July 2001, EMSCI is a prospective longitudinal cohort study conforming to the standards established by the Declaration of Helsinki and approved by the local ethics committees of all participating centres. Before entering the study, patients were thoroughly informed about the study procedures and provided written informed consent. Data for neurological and functional assessments were prospectively collected per protocol within the first 15 d (very acute), between 16–40 d (acute I), and 3 mo (acute II), 6 mo (acute III), and 12 mo (chronic) after SCI (Fig 1). From the EMSCI database, we extracted the data of all patients with a date of traumatic SCI between July 2001 and December 2012.

Our report conforms to the transparent reporting of a multivariable prediction model for individual prognosis or diagnosis (TRIPOD) statement (http://www.equator-network.org/reporting-guidelines/tripod-statement/) (S1 Text).

### Neurourological Management

All patients entered a rehabilitation program that included bladder and bowel management. The neurourological management was patient tailored and according to the EAU Guidelines on Neuro-Urology [8]. In brief, based on urodynamic investigation, the appropriate therapeutic strategy in order to preserve both upper and lower urinary tract function was determined. In patients with detrusor overactivity, the concept was to convert the overactive detrusor into a normo- or underactive detrusor using antimuscarinics or intradetrusor onabotulinumtoxinA injections in a refractory situation. Patients with voiding dysfunction due to an underactive/acontractile detrusor and/or detrusor sphincter dyssynergia generally relied on intermittent self-catheterization, but some patients who were not able to do so were managed using an indwelling transurethral or suprapubic catheter.

### Predictive Variables

As possible predictive variables (S1 and S2 Data), we investigated patients’ age and sex, all data derived from the neurological examination according to the International Standards for Neurological Classification of Spinal Cord Injury (ISNCSCI) [17–19] and from the functional assessment according to the Spinal Cord Independence Measure (SCIM) versions II and III [20,21]. The ISNCSCI is an established neurological assessment developed and published by American Spinal Injury Association (ASIA) to determine the level and classify the severity of SCI [17–19]. This grading system rates with a six-point scale (from 0 = total paralysis to 5 = active movement with full range of motion against gravity and full resistance) the muscle strength of ten key muscle groups of upper and lower limbs for each side and records the light-touch and pinprick sensation quality for each dermatome of the body (0 = absent, 1 = impaired, 2 = normal) and the presence of voluntary anal contraction and sensation of deep anal pressure. The ISNCSCI provides some cumulative scores, i.e., upper extremity motor score (UEMS), lower extremity motor score (LEMS), and light-touch and pinprick scores, and allows the definition of neurological level, motor level, and sensory level and classification with the ASIA Impairment Scale (AIS) in five different grades of severity (from A = complete lesion to E = normal sensation and motor function in all segments).

ISNCSCI assessments were performed by trained physicians with certified experience in SCI examination and classification, after a specific centralized training program [22]. Motor and sensory scores and AIS grades were computed automatically by the EMSCI’s ISNCSCI calculator (www.ais.emsci.org).

The SCIM is a validated tool specifically designed for the assessment of functional capacity of patients affected by SCI and investigates the ability to perform SCI-relevant tasks of daily life activity, clustered into three subscales: the self-care domain (with a score range of 0–20), including feeding, bathing, dressing, and grooming; respiration and sphincter management (score range 0–40), including respiration, bladder, and bowel management and use of toilet; and mobility (score range 0–40), including mobility in bed, transfers, mobility indoors and outdoors, and stair management. The total SCIM score ranges between 0 and 100, with higher scores reflecting higher levels of independence. Over the last years, two revisions of this instrument have been proposed, SCIM version II and III, respectively [20,21]. SCIM version II and III show minor differences in item organization and scoring but have the same maximum scores within the three subscales and concerning total score calculation.

EMSCI initially applied SCIM version II and switched to version III after its introduction. The version used in the initial assessment of each patient was employed for further follow-up evaluations. SCIM assessments were performed by health professionals specially trained and experienced in the use of this tool.

All predictive variables have been recorded within the first 40 d from injury (Fig 1). When available, the acute I assessment (16 to 40 d after injury) has been chosen (*n* = 1,195). When those data were missing, the very acute measurement (within 15 d from injury) was considered (*n* = 55).

### Outcome Measure

The primary outcome was urinary continence (assessed by bladder diary) and complete bladder emptying (i.e., postvoid residual < 100 mL assessed by ultrasound or “in-out” catheterization) 1 y after SCI (time point chronic, Fig 1) and measured through item 6 (sphincter management—bladder) of SCIM [20,21].

Patients were dichotomized on the basis of bladder function 1 y after SCI into (a) urinary continence and complete bladder emptying when rated with the maximum item 6 score (i.e., 15 points) or (b) urinary incontinence and/or voiding dysfunction of various degrees when displaying a lower score (i.e., item 6 score < 15 points).

### Statistical Analysis

Based on an earlier publication derived from the same database, which included patients from July 2001 until June 2008 [15], we anticipated a substantial number of missing data in the outcome. We therefore used a weighting approach to correct for missing data. In this method, complete cases are weighted by the inverse probability of being a complete case [23]. The most relevant factors associated with missing outcome data were centre, year of inclusion, and age. Thus, we calculated the probability of missing data based on these parameters and defined a weight (w) = 1/1 − probability of missing.

Missing data for predictor parameters were rare (no missing data on the SCIM scores and < 5% on the ISNCSCI impairment scale).

Data derived from ISNCSCI concerning motor and sensory levels as well as any single and cumulative sensory and motor score for each body side have been transformed from “right” and “left” into “best” and “worst.”

In 80 patients, missing data concerning S4–S5 dermatome sensation, deep anal pressure, and/or voluntary anal contraction have been reconstructed on the basis of S1 function, as proposed by Zariffa et al. [24], allowing the determination of AIS grade.

All 182 available covariates were used. Based on the Akaike information criterion and using a stepwise forward procedure, we selected the potential predictors. No interactions were considered. At each step, the area under the receiver operating characteristics curve (aROC) was calculated. The procedure was stopped if the aROC did not increase significantly (*p* ≥ 0.05).

Patients’ characteristics were reported as percentage or mean (standard deviation), and comparisons were performed using parametric and nonparametric tests, as appropriate. Statistical analyses (S2 Text) were performed using the R statistics package (R version 2.14.0, www.R-project.org/).

### Validation Study

To evaluate the predictive power of our models in an independent clinical dataset, we retrospectively collected data of patients affected by traumatic SCI, which were referred to the Spinal Cord Rehabilitation Unit of Santa Lucia Foundation, Rome, Italy (S3 Data). This centre has been included in the EMSCI network since February 2013. For the validation of our models, we considered all patients with traumatic SCI evaluated within 40 d of the injury, fulfilling the inclusion and exclusion criteria of EMSCI from January 2005 to January 2013. The physicians who performed the selection of patients and data collection were blinded to the prediction model characteristics.

## Results

### Patients

In 18 EMSCI centres, a total of 2,269 patients with traumatic SCI were enrolled between July 2001 and December 2012. Of those, the outcome measure 1 y after injury was available in 1,331 patients, but the initial ISNCSCI assessment was missing in 81 patients, leaving the data of 1,250 patients for the prediction analysis. The clinical characteristics at inclusion for patients considered for the model derivation and for those missing 1-y follow-up are reported in Table 1. There were significant differences between the two groups regarding age, percentage of paraplegics, percentage of patients with complete lesions (AIS A), and SCIM total score.

**Table 1:** Characteristics of patients at the time of inclusion (within 40 d from spinal cord injury).

	EMSCI Cohort				External Cohort		
	Lost at Follow-up	*p*-Value	Derivation	*p*-Value	Validation	*p*-Value	Lost at Follow-up
	(*n* = 938)	↔	(*n* = 1,250)	↔	(*n* = 111)	↔	(*n* = 24)
Age, y: mean (SD)	46.5 (19.2)	<0.001	42.5 (17.6)	<0.001	40.0 (16.1)	<0.001	46.1 (16.1)
Sex: *n* (%) of males	733 (78.1%)	0.383	996 (79.7%)	0.002	102 (91.9%)	0.493	21 (87.5%)
**Neurological Level** ¶							
C1–C8: *n* (%)	494 (53.0%)	0.092	617 (49.4%)	0.075	45 (40.5%)	0.154	6 (25.0%)
T1–T12: *n* (%)	300 (32.2%)	0.058	451 (36.1%)	0.001	57 (51.4%)	0.390	10 (41.7%)
L1–L5: *n* (%)	97 (10.4%)	0.321	147 (11.8%)	0.148	8 (7.2%)	0.010	6 (25.0%)
S1–S5: *n* (%)	4 (0.4%)	0.412*	2 (0.2%)	0.225*	1 (0.9%)	0.325*	1 (4.2%)
Not testable: *n* (%)	37 (4.0%)	0.081	33 (2.6%)		-		1 (4.2%)
**Plegia**							
Tetraplegia: *n* (%)	407 (43.4%)	0.101	610 (48.8%)	0.042	43 (38.7%)	0.379	7 (29.2%)
Paraplegia: *n* (%)	491 (52.4%)	0.032	600 (48%)	0.007	68 (61.3%)	0.379	17 (70.8%)
Not testable: *n* (%)	40 (4.3%)	0.189	40 (3.2%)		-		-
**Severity of Neurological Deficit** ¶							
AIS A: *n* (%)	376 (40.1%)	0.040	556 (44.5%)	0.013	63 (56.8%)	0.014	7 (29.2%)
AIS B: *n* (%)	98 (10.5%)	0.576	140 (11.2%)	0.901	12 (10.8%)	0.124*	0
AIS C: *n* (%)	151 (16.1%)	0.638	192 (15.4%)	0.319	12 (10.8%)	0.180	5 (20.8%)
AIS D: *n* (%)	275 (29.3%)	0.294	341 (27.3%)	0.197	24 (21.6%)	0.004	12 (50.0%)
AIS E: *n* (%)	6 (0.6%)	0.184*	3 (0.2%)	0.999*	0	-	0
Not testable: *n* (%)	32 (3.4%)	0.002	18 (1.4%)		-		-
LEMS ¶: mean (SD)	16.1 (18.8)	0.020	14.2 (18.1)	0.018	10.2 (16.7)	0.005	23.0 (19.4)
Urinary continence and complete bladder emptying: *n* (%)	117 (12.5%)	0.653	148 (11.9%)	0.008*	4 (3.6%)	<0.001*	7 (29.2%)
SCIM total score: mean (SD)	30.4 (25.5)	0.830	30.1 (23.7)	<0.001	17.7 (12.0)	0.007	35.8 (29.4)

*Fisher’s exact test (two-sided).

^¶^ Based on International Standards for Neurological Classification of Spinal Cord Injury (ISNCSCI).

Abbreviations: SD, standard deviation; EMSCI, European Multicenter Spinal Cord Injury study; AIS, American Spinal Injury Association Impairment Scale; LEMS, lower extremity motor score; SCIM, Spinal Cord Independence Measure

In the derivation group, urinary continence and complete bladder emptying were found in 148 (12%) patients at initial assessment and remained unchanged at the 1-y follow-up in all but two of these patients.

One year after SCI, 398 of the 1,250 patients (32%: 91 of 254 females [36%] and 307 of 996 males [31%]) showed urinary continence and complete bladder emptying. Among those, 91 (23%) were females, and 218 (55%) were tetraplegics. Of 852 patients with urinary incontinence and/or incomplete bladder emptying, 163 (19%) were females, and 392 (46%) were tetraplegics.

### Full Model

**Fig 2:**
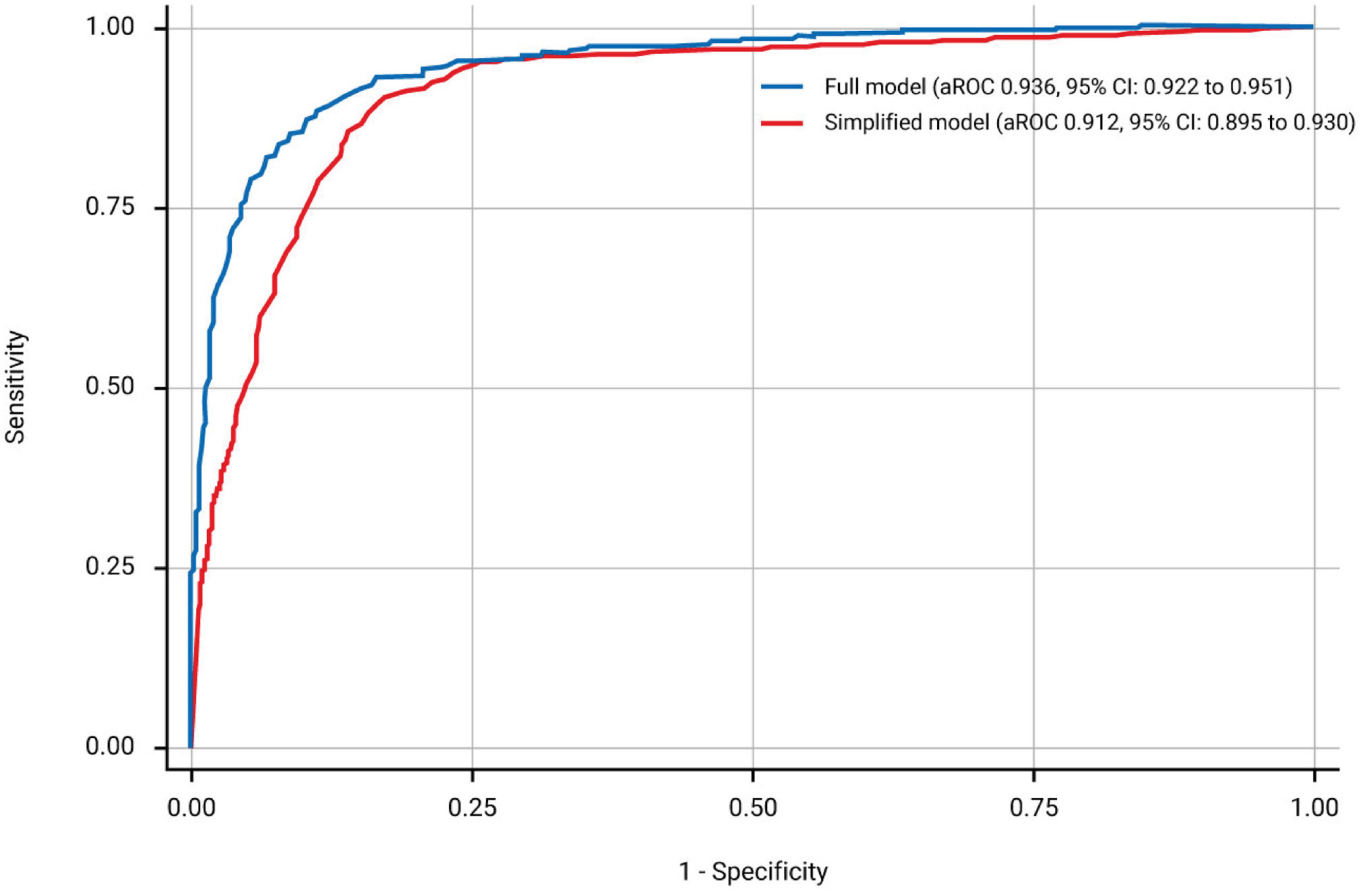
Receiver operating characteristics curves and corresponding area under the receiver operating characteristics curve (aROC) of the two models. The full model (blue line) is based on the lower extremity motor score (LEMS), the highest score between right and left side light-touch sensation in the S3 dermatome of International Standards for Neurological Classification of Spinal Cord Injury (ISNCSCI), and the respiration and sphincter management subscale score of the Spinal Cord Independence Measure (SCIM). The simplified model (red line) relies on LEMS only.

The full model for prediction of urinary continence and complete bladder emptying contained three predictors: LEMS of ISNCSCI (aROC = 0.912 [95% CI: 0.895–0.930]), the highest score between right and left side of the light-touch sensation in the S3 dermatome of ISNCSCI (aROC = 0.927 [0.911–0.942]; aROC significant increase, *p* < 0.001), and SCIM subscale respiration and sphincter management (aROC significant increase, *p* = 0.001). The aROC of the final model was 0.936 (95% CI: 0.922–0.951)—see Fig 2, S1 Table, the calibration plot in S1 Fig and S2 Table. The complete function along with an example is shown in S4 Data.

When applying the model in sensitivity analyses after excluding the 55 patients with very acute measurements only and the 148 patients with urinary continence and complete bladder emptying at inclusion, the aROCs were 0.934 (95% CI: 0.918–0.949) and 0.909 (95% CI: 0.889–0.930), respectively.

### Simplified Model

**Fig 3:**
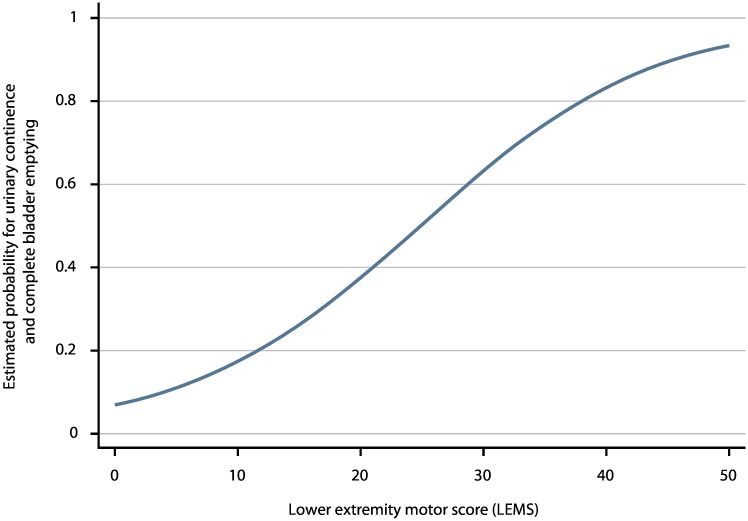
Estimated probabilities for urinary continence and complete bladder emptying based on LEMS values. LEMS values of ISNCSCI within 40 d after spinal cord injury (SCI) and the corresponding estimated probabilities for urinary continence and complete bladder emptying 1 y after SCI.

As the aROC of a model with the best predictor, i.e., LEMS, was comparable with the aROC of the full model, we defined this as the simple model. The aROC was 0.912 (95% CI: 0.895–0.930)—see Fig 2, S3 Table, and the calibration plot in S2 Fig. When applying the model after excluding the 55 patients with very acute measurements and the 148 patients with urinary continence and complete bladder emptying at inclusion, the aROCs were 0.908 (95% CI: 0.890–0.926) and 0.889 (95% CI: 0.867–0.911). The relationship of LEMS values at the time of inclusion and corresponding estimated probabilities for urinary continence and complete bladder emptying is shown in Fig 3 and Table 2.

**Table 2:** Estimated probabilities for urinary continence and complete bladder emptying 1 y after SCI based on LEMS value within 40 d.

LEMS Value	Estimated Probabilities
0	6.7%
1	7.4%
2	8.1%
3	8.9%
4	9.8%
5	10.8%
6	11.9%
7	13.0%
8	14.3%
9	15.6%
10	17.1%
11	18.6%
12	20.3%
13	22.1%
14	23.9%
15	25.9%
16	28.0%
17	30.2%
18	32.5%
19	34.8%
20	37.3%
21	39.8%
22	42.4%
23	45.0%
24	47.6%
25	50.2%
26	52.9%
27	55.5%
28	58.1%
29	60.7%
30	63.2%
31	65.6%
32	67.9%
33	70.2%
34	72.4%
35	74.4%
36	76.4%
37	78.3%
38	80.0%
39	81.6%
40	83.2%
41	84.6%
42	85.9%
43	87.2%
44	88.3%
45	89.4%
46	90.3%
47	91.2%
48	92.0%
49	92.8%
50	93.5%

### External Validation

Data of 135 patients with traumatic SCI were collected. The outcome measure 1 y after injury was available for 111 patients, who were considered for external validation. Table 1 shows the patients’ characteristics of the external cohort. At the time of inclusion, there were differences between the validation and lost at 1-y follow-up groups regarding age, neurological level, severity of neurological deficit, LEMS, urinary continence and complete bladder emptying, and SCIM total score. The validation versus derivation cohort was significantly different: patients of the validation cohort were younger, more often men, and paraplegics. In addition, they were more severely affected, i.e., more patients with complete lesion and impaired bladder function, lower LEMS, and lower SCIM total score.

At initial assessment, four (4%) patients displayed urinary continence and complete bladder emptying, and all remained unchanged at 1-y follow-up. One year after SCI, 27 (24%) patients showed urinary continence and complete bladder emptying. Among those, four (4%) were females, and 15 (14%) were tetraplegics.

External validation of the full and simplified model confirmed excellent predictive power: 0.965 (95% CI: 0.934–0.996) and 0.972 (95% CI 0.943–0.999), respectively.

## Discussion

### Principal Findings

In the present study, we derived two reliable prediction models with excellent performance to estimate the probability of urinary continence and complete bladder emptying 1 y after traumatic SCI. The full model integrates three simple clinical parameters derived from ISNCSCI and SCIM: LEMS (i.e., the sum of the motor scores of the five lower extremity key muscle groups on both sides: hip flexors, knee extensors, ankle dorsiflexors, long toe extensors, and ankle plantar flexors), light-touch sensation of the S3 dermatome (i.e., the highest score between the right and left side for S3 dermatome light-touch assessment), and the SCIM subscale respiration and sphincter management (i.e., assessment of independence in breathing, bladder and bowel management, and use of toilet). Also, the simplified model retains an excellent predictive performance and is easier and faster to apply in daily clinical practice. It exclusively relies on LEMS, which is part of routine neurological assessment of patients with SCI, and introduces a very simple, rapid, noninvasive, and inexpensive tool to predict urinary continence and complete bladder emptying 1 y after injury without the need of any specific equipment.

In addition, our findings indicate that patients with urinary continence and complete bladder emptying within 40 d after SCI are very likely to maintain these functions 1 y after injury and that approximately one-third of all patients with traumatic SCI will have favourable bladder outcomes after 1 y.

### Findings in the Context of the Literature

Based on a large multicentre United States database [25] and on the findings of the present study on a European population, the reported rate of patients with urinary continence and complete bladder emptying 1 y after traumatic SCI ranges from 27% to 36% of females and from 21% to 31% of male patients. Over the last years, only a few studies on limited numbers of patients have investigated the role of clinical and neurophysiological parameters, and they have not found reliable predictors of bladder function [12,26,27]: in 28 patients with SCI, perianal (S4–S5) pinprick sensation and bulbocavernosus (S2–S4) reflex were moderately sensitive in predicting the return of spontaneous voiding but could not predict detrusor overactivity and detrusor sphincter dyssynergia [12]. AIS and somatosensory evoked potentials were not indicative for recovery of bladder function in 70 patients with SCI [26]. In addition, neurogenic bladder dysfunction could not be predicted by sensory evaluation in 55 patients with SCI due to thoracolumbar fractures [27]. Importantly, from a neurophysiological viewpoint, bladder function largely relies on the integrity of the autonomous system [28], while our models mainly include predictors controlled by the somatic nervous system. Thus, correlation of our models with urodynamic and neurophysiological data might further enhance our understanding of different clinical patterns and evolution of neurogenic urinary tract dysfunction after SCI, warranting further prospective investigations.

Early initiation of rehabilitation after SCI is mandatory, and the definition of functional prognosis plays a key role in establishing the rehabilitative goals [12,13]. In 2009, the Spinal Cord Outcomes Partnership Endeavor (SCOPE) identified SCIM III as a primary outcome measure of functional recovery for patients with SCI (e.g., as a primary outcome measure for pivotal phase III clinical trials) and encouraged its wide diffusion [29]. SCIM was already chosen as an outcome measure for the prediction models of locomotion [15] and upper limb function [16], and the presence of a specific item on bladder function makes this tool ideal for the assessment of urological function after SCI.

Based on our findings, it cannot be argued that LEMS could replace standard neurourological evaluation, since our models are derived from data of patients managed according to the EAU Guidelines on Neuro-Urology [8] implying that even in patients with high probability of positive bladder outcomes based on LEMS, complete neurourological evaluation including urodynamic investigation remains mandatory for an optimal neurourological management. Indeed, in patients with SCI, videourodynamics, a combination of cystometry and pressure-flow study (in those who are able to void) with simultaneous fluoroscopic monitoring assessing detrusor and bladder outlet function and providing information about detrusor pressure and compliance—and thus, the risk factors for upper urinary tract damage—is essential for clinical decision making [8,9].

### Strengths and Limitations

Our prediction models were developed using data obtained from a large population of patients with traumatic SCI prospectively enrolled in the EMSCI. All variables included in the models were derived from ISNCSCI and SCIM, which are routinely employed in European SCI centres. Our simplified prediction model is straightforward, not requiring any specific equipment, thereby allowing its easy application in the daily clinical setting.

The excellent predictive power of our models was confirmed by external validation, thereby further extending and strengthening their validity [30]. The patients of the validation differed from the derivation cohort in several characteristics and were more severely affected, which might have contributed to the slightly higher predictive power of our models in the validation compared with the derivation cohort. Differences in patients’ characteristics between the two cohorts were most probably due to a centre effect (single centre versus multicentre) and data collection modality (retrospective versus prospective).

The major limitation of our study lies in the substantial number of patients with a missing 1-y outcome and thus the possibility of selection bias. However, the group of patients without 1-y follow-up showed only minor differences in patient characteristics compared with the derivation group. That the model performed well in the validation cohort reassures us that selection bias may not be an issue. Moreover, the effect of missing data is taken into account and limited by the weighting approach used in our analysis. In addition, although neurourological management was according to the generally accepted EAU Guidelines on Neuro-Urology [8], we did not assess the effect of treatment on bladder outcomes. Thus, our findings have to be seen under the prerequisite that the EAU Guidelines on Neuro-Urology [8] are followed.

The finding that complete bladder function within 40 d does not deteriorate at 1 y was previously unknown. Therefore, we included in the derivation model all the patients of the sample and also those with a complete bladder function at inclusion. We then confirmed the high predictive power of our models after excluding patients with complete bladder function at inclusion.

### Implications for Practice and Research

The use of our prediction models could allow early identification of the about two-thirds of patients who are not likely to show urinary continence and complete bladder emptying 1 y after SCI despite state-of-the-art treatment. This identification would be highly desirable to improve counselling and early orient an individualized urological management, with positive consequences on both the level of care and funding allocation. Patients who are unlikely to recover a complete bladder function could take advantage from early introduction of specific rehabilitative interventions, such as neuromodulative procedures [12,13]. Indeed, early bilateral sacral neuromodulation during the early phase of SCI prevented the development of neurogenic detrusor overactivity and urinary incontinence and also improved erectile and bowel dysfunction in patients with complete SCI [31]. However, long-term results are pending, and the exact mechanism of action is not well understood. Nevertheless, other neuromodulation techniques such as tibial nerve stimulation [32] and transcutaneous electrical nerve stimulation [33] might also be promising, warranting well-designed randomized-controlled trials.

The urological management and the treatment of systemic complications of neurogenic bladder dysfunction are accountable for a conspicuous part of the huge direct and indirect medical expenses for patients with SCI, and the early optimization of patient-tailored treatment could dramatically reduce these costs [34].

The introduction of our models could also positively impact on the design of future neurourological clinical trials for SCI. In particular, our models could allow for specifically enrolling patients less likely to achieve a complete bladder function based on standard therapies as well as balancing the different treatment groups based on the predicted probability of bladder function recovery.

### Conclusions

Our study provides two simple and reliable models to predict urinary continence and complete bladder emptying 1 y after traumatic SCI. The simplified prediction rule exclusively relies on LEMS, which is part of routine neurological assessment of patients with SCI, and introduces a very simple, rapid, noninvasive, and inexpensive tool that can be used without the need of any specific equipment. This model can be easily employed in daily clinical practice for early counselling and orientation of patient-tailored rehabilitative interventions, resulting in a higher level of care, and it might improve patient stratification in future clinical studies.

## Supporting Information

S1 Data Methods. List of variables evaluated as possible predictors.
(DOCX)

S2 Data Methods. Data of the derivation cohort.
(XLSX)

S3 Data Methods. Data of the validation cohort.
(XLSX)

S4 Data Results. Full model for prediction of urinary continence and complete bladder emptying.
(DOCX)

S1 Fig Results. Calibration plot of the full model.
(EPS)

S2 Fig Results. Calibration plot of the simplified model.
(EPS)

S1 Table Results. Cut-off values of the full model in the derivation cohort and the corresponding sensitivity and specificity.
(DOCX)

S2 Table Results. The table shows how the additional parameters were associated with increases of the aROC. *p*-Values indicate comparisons between the aROCs from model 1 versus model 2 and model 2 versus model 3, respectively.
(DOCX)

S3 Table Results. Cut-off values of the simplified model in the derivation cohort and the corresponding sensitivity and specificity.
(DOCX)

S1 Text Methods. TRIPOD statement checklist.
(DOCX)

S2 Text Methods. Data analysis plan.
(DOCX)

## Abbreviations

aROC: area under the receiver operating characteristics curve
AIS: American Spinal Injury Association Impairment Scale
ASIA: American Spinal Injury Association
CI: confidence interval
EAU: European Association of Urology
EMSCI: European Multicenter Study about Spinal Cord Injury
ISNCSCI: International Standards for Neurological Classification of Spinal Cord Injury
LEMS: lower extremity motor score
SCI: spinal cord injury
SCIM: Spinal Cord Independence Measure
SCOPE: Spinal Cord Outcomes Partnership Endeavor
TRIPOD: transparent reporting of a multivariable prediction model for individual prognosis or diagnosis
UEMS: upper extremity motor score
UTI: urinary tract infection
