# Supplementary material for: Prediction of Bladder Outcomes after Traumatic Spinal Cord Injury: A Longitudinal Cohort Study
Source: PLoS Med. 2016 Jun 21;13(6):e1002041. doi: 10.1371/journal.pmed.1002041 (PMC4915662; doi:10.1371/journal.pmed.1002041)
Supplement: S1 Data — List of variables evaluated as possible predictors. (DOCX) [file pmed.1002041.s001.docx]

**S1 Data**

List of variables evaluated as possible predictors

- Sex
- Age at injury
- Year of injury
- SCIM subscore Self-Care
- SCIM subscore Respiration and Sphincter Management
- SCIM subscore Mobility
- SCIM subscore Mobility (room and toilet)
- SCIM subscore Mobility (indoors and outdoors)
- SCIM total score
- SCIM item 1
- SCIM item 2a
- SCIM item 2b
- SCIM item 3a
- SCIM item 3b
- SCIM item 4
- SCIM item 5
- SCIM item 6
- SCIM item 7
- SCIM item 8
- SCIM item 9
- SCIM item 10
- SCIM item 11
- SCIM item 12
- SCIM item 13
- SCIM item 14
- SCIM item 15
- SCIM item 16
- SCIM item 17
- ASIA Impairment Scale (AIS)
- Neurological level
- Motor level best
- Motor level worst
- Sensory level best
- Sensory level worst
- Plegia (tetraplegia or paraplegia)
- Total motor score
- Upper extremity motor score (UEMS)
- Upper extremity motor score best
- Upper extremity motor score worst
- Lower extremity motor score (LEMS)
- Lower extremity motor score best
- Lower extremity motor score worst
- Motor score C5 best
- Motor score C6 best
- Motor score C7 best
- Motor score C8 best
- Motor score T1 best
- Motor score L2 best
- Motor score L3 best
- Motor score L4 best
- Motor score L5 best
- Motor score S1 best
- Motor score C5 worst
- Motor score C6 worst
- Motor score C7 worst
- Motor score C8 worst
- Motor score T1 worst
- Motor score L2 worst
- Motor score L3 worst
- Motor score L4 worst
- Motor score L5 worst
- Motor score S1 worst
- Voluntary anal contraction
- Total Pin Prick Sensory score
- Pin Prick Sensory best
- Pin Prick Sensory worst
- Pin Prick Sensory C2 best
- Pin Prick Sensory C3 best
- Pin Prick Sensory C4 best
- Pin Prick Sensory C5 best
- Pin Prick Sensory C6 best
- Pin Prick Sensory C7 best
- Pin Prick Sensory C8 best
- Pin Prick Sensory T1 best
- Pin Prick Sensory T2 best
- Pin Prick Sensory T3 best
- Pin Prick Sensory T4 best
- Pin Prick Sensory T5 best
- Pin Prick Sensory T6 best
- Pin Prick Sensory T7 best
- Pin Prick Sensory T8 best
- Pin Prick Sensory T9 best
- Pin Prick Sensory T10 best
- Pin Prick Sensory T11 best
- Pin Prick Sensory T12 best
- Pin Prick Sensory L1 best
- Pin Prick Sensory L2 best
- Pin Prick Sensory L3 best
- Pin Prick Sensory L4 best
- Pin Prick Sensory L5 best
- Pin Prick Sensory S1 best
- Pin Prick Sensory S2 best
- Pin Prick Sensory S3 best
- Pin Prick Sensory S4-5 best
- Pin Prick Sensory C2 worst
- Pin Prick Sensory C3 worst
- Pin Prick Sensory C4 worst
- Pin Prick Sensory C5 worst
- Pin Prick Sensory C6 worst
- Pin Prick Sensory C7 worst
- Pin Prick Sensory C8 worst
- Pin Prick Sensory T1 worst
- Pin Prick Sensory T2 worst
- Pin Prick Sensory T3 worst
- Pin Prick Sensory T4 worst
- Pin Prick Sensory T5 worst
- Pin Prick Sensory T6 worst
- Pin Prick Sensory T7 worst
- Pin Prick Sensory T8 worst
- Pin Prick Sensory T9 worst
- Pin Prick Sensory T10 worst
- Pin Prick Sensory T11 worst
- Pin Prick Sensory T12 worst
- Pin Prick Sensory L1 worst
- Pin Prick Sensory L2 worst
- Pin Prick Sensory L3 worst
- Pin Prick Sensory L4 worst
- Pin Prick Sensory L5 worst
- Pin Prick Sensory S1 worst
- Pin Prick Sensory S2 worst
- Pin Prick Sensory S3 worst
- Pin Prick Sensory S4-5 worst
- Total Light Touch Sensory score
- Light Touch Sensory best
- Light Touch Sensory worst
- Light Touch Sensory C2 best
- Light Touch Sensory C3 best
- Light Touch Sensory C4 best
- Light Touch Sensory C5 best
- Light Touch Sensory C6 best
- Light Touch Sensory C7 best
- Light Touch Sensory C8 best
- Light Touch Sensory T1 best
- Light Touch Sensory T2 best
- Light Touch Sensory T3 best
- Light Touch Sensory T4 best
- Light Touch Sensory T5 best
- Light Touch Sensory T6 best
- Light Touch Sensory T7 best
- Light Touch Sensory T8 best
- Light Touch Sensory T9 best
- Light Touch Sensory T10 best
- Light Touch Sensory T11 best
- Light Touch Sensory T12 best
- Light Touch Sensory L1 best
- Light Touch Sensory L2 best
- Light Touch Sensory L3 best
- Light Touch Sensory L4 best
- Light Touch Sensory L5 best
- Light Touch Sensory S1 best
- Light Touch Sensory S2 best
- Light Touch Sensory S3 best
- Light Touch Sensory S4-5 best
- Light Touch Sensory C2 worst
- Light Touch Sensory C3 worst
- Light Touch Sensory C4 worst
- Light Touch Sensory C5 worst
- Light Touch Sensory C6 worst
- Light Touch Sensory C7 worst
- Light Touch Sensory C8 worst
- Light Touch Sensory T1 worst
- Light Touch Sensory T2 worst
- Light Touch Sensory T3 worst
- Light Touch Sensory T4 worst
- Light Touch Sensory T5 worst
- Light Touch Sensory T6 worst
- Light Touch Sensory T7 worst
- Light Touch Sensory T8 worst
- Light Touch Sensory T9 worst
- Light Touch Sensory T10 worst
- Light Touch Sensory T11 worst
- Light Touch Sensory T12 worst
- Light Touch Sensory L1 worst
- Light Touch Sensory L2 worst
- Light Touch Sensory L3 worst
- Light Touch Sensory L4 worst
- Light Touch Sensory L5 worst
- Light Touch Sensory S1 worst
- Light Touch Sensory S2 worst
- Light Touch Sensory S3 worst
- Light Touch Sensory S4-5 worst
- Deep anal pressure
