# Supplementary material for: Prediction of Bladder Outcomes after Traumatic Spinal Cord Injury: A Longitudinal Cohort Study
Source: PLoS Med. 2016 Jun 21;13(6):e1002041. doi: 10.1371/journal.pmed.1002041 (PMC4915662; doi:10.1371/journal.pmed.1002041)
Supplement: S4 Data — Full model for prediction of urinary continence and complete bladder emptying. (DOCX) [file pmed.1002041.s004.docx]

**S4 Data**

Full model for prediction of urinary continence and complete bladder emptying.

The specific probability (P) of urinary continence and complete bladder emptying was computed using the following formula:

P = 1/[1 + exp(−S)]

involving S= -4.392515 + 1.412322x1 +0.0623388x2 +0.068302x3

where:

x1= Highest score between right and left side for S3 dermatome light-touch assessment [0, 1, 2]

x2= Lower extremity motor score (LEMS) [0 – 50]

x3= SCIM subscale respiration and sphincter management [0-40]

For example: a patient with the highest score between right and left side for S3 dermatome light-touch assessment of 1, a LEMS value of 25 and a SCIM subscale respiration and sphincter management value of 7 would results in:

S= -4.392515 + 1.412322 +25*0.0623388 + 22*0.068302 = 0.080921

P= 1/[1 + e-0.080921]= 1/[1 + 0.922267] = 52.0219 %
