# Supplementary material for: Prediction of Bladder Outcomes after Traumatic Spinal Cord Injury: A Longitudinal Cohort Study
Source: PLoS Med. 2016 Jun 21;13(6):e1002041. doi: 10.1371/journal.pmed.1002041 (PMC4915662; doi:10.1371/journal.pmed.1002041)
Supplement: S1 Table — Cut-off values of the full model in the derivation cohort and the corresponding sensitivity and specificity. (DOCX) [file pmed.1002041.s007.docx]

**S1 Table**

Cut-off values of the full model in the derivation cohort and corresponding sensitivity and specificity

| Positive if Greater Than or Equal To ^a^ | Sensitivity | 1 - Specificity |
| --- | --- | --- |
| -,8763 | 1,000 | 1,000 |
| ,1729 | 1,000 | ,929 |
| ,3015 | 1,000 | ,917 |
| ,5137 | 1,000 | ,905 |
| ,7574 | 1,000 | ,893 |
| ,8718 | 1,000 | ,310 |
| ,8820 | 1,000 | ,274 |
| ,9026 | 1,000 | ,262 |
| ,9213 | ,963 | ,262 |
| ,9461 | ,963 | ,250 |
| ,9683 | ,963 | ,190 |
| ,9728 | ,926 | ,167 |
| ,9774 | ,926 | ,155 |
| ,9840 | ,889 | ,155 |
| ,9888 | ,889 | ,119 |
| ,9898 | ,852 | ,107 |
| ,9912 | ,815 | ,107 |
| ,9936 | ,815 | ,095 |
| ,9957 | ,778 | ,095 |
| ,9963 | ,741 | ,083 |
| ,9966 | ,704 | ,083 |
| ,9970 | ,704 | ,071 |
| ,9976 | ,704 | ,048 |
| ,9980 | ,667 | ,048 |
| ,9982 | ,630 | ,048 |
| ,9985 | ,593 | ,048 |
| ,9986 | ,593 | ,036 |
| ,9987 | ,556 | ,036 |
| ,9990 | ,519 | ,036 |
| ,9993 | ,481 | ,036 |
| ,9996 | ,444 | ,036 |
| ,9998 | ,407 | ,036 |
| ,9999 | ,407 | ,024 |
| 1,0000 | ,370 | ,024 |
| 1,0000 | ,370 | ,012 |
| 1,0000 | ,333 | ,012 |
| 1,0000 | ,296 | ,012 |
| 1,0000 | ,259 | ,012 |
| 1,0000 | ,259 | ,000 |
| 1,0000 | ,222 | ,000 |
| 1,0000 | ,148 | ,000 |
| 2,0000 | ,000 | ,000 |
|  | | |
