# Supplementary material for: Prediction of Bladder Outcomes after Traumatic Spinal Cord Injury: A Longitudinal Cohort Study
Source: PLoS Med. 2016 Jun 21;13(6):e1002041. doi: 10.1371/journal.pmed.1002041 (PMC4915662; doi:10.1371/journal.pmed.1002041)
Supplement: S2 Table — The table shows how the additional parameters were associated with increases of the aROC. p-Values indicate comparisons between the aROCs from model 1 versus model 2 and model 2 versus model 3, respectively. (DOCX) [file pmed.1002041.s008.docx]

**S2 Table**

The table shows how the additional parameters were associated with increases of the aROC. P-values indicate comparisons between the aROCs from model 1 vs. 2 and model 2 vs. model 3 respectively.

| **Model** | **Parameter** | **aROC (95% CI)** | **p-value** |
| --- | --- | --- | --- |
| 1 | Lower extremity motor score (LEMS) | 0**.**912  (0**.**895 to 0**.**930) |  |
| 2 | + highest score between right and left side of the light-touch sensation in S3 dermatome | 0.927 (0.911 to 0.942) | Comparison 1 vs. 2: p<0.001 |
| 3 | + SCIM subscale respiration and sphincter management | 0.936  (0.922 to 0.951) | Comparison 2 vs. 3: p=0.001 |
