# Supplementary material for: Prediction of Bladder Outcomes after Traumatic Spinal Cord Injury: A Longitudinal Cohort Study
Source: PLoS Med. 2016 Jun 21;13(6):e1002041. doi: 10.1371/journal.pmed.1002041 (PMC4915662; doi:10.1371/journal.pmed.1002041)
Supplement: S3 Table — Cut-off values of the simplified model in the derivation cohort and the corresponding sensitivity and specificity. (DOCX) [file pmed.1002041.s009.docx]

**S3 Table**

Cut-off values of the simplified model in the derivation cohort and corresponding sensitivity and specificity

| Positive if Greater Than or Equal To^a^ | Sensitivity | 1 - Specificity |
| --- | --- | --- |
| ,00000 | 1,000 | 1,000 |
| ,06100 | 1,000 | ,190 |
| ,06748 | 1,000 | ,143 |
| ,07459 | ,963 | ,119 |
| ,08683 | ,963 | ,107 |
| ,11126 | ,963 | ,095 |
| ,14011 | ,926 | ,095 |
| ,16033 | ,926 | ,071 |
| ,23372 | ,889 | ,060 |
| ,32343 | ,852 | ,036 |
| ,39847 | ,815 | ,036 |
| ,50365 | ,778 | ,036 |
| ,58354 | ,741 | ,036 |
| ,62239 | ,704 | ,036 |
| ,67068 | ,630 | ,036 |
| ,72764 | ,593 | ,024 |
| ,75884 | ,556 | ,024 |
| ,77801 | ,556 | ,012 |
| ,79607 | ,444 | ,012 |
| ,84045 | ,407 | ,012 |
| ,88171 | ,296 | ,012 |
| ,89250 | ,148 | ,012 |
| ,90676 | ,074 | ,012 |
| ,91982 | ,037 | ,000 |
| 1,00000 | ,000 | ,000 |
