# Supplementary material for: Prediction of Bladder Outcomes after Traumatic Spinal Cord Injury: A Longitudinal Cohort Study
Source: PLoS Med. 2016 Jun 21;13(6):e1002041. doi: 10.1371/journal.pmed.1002041 (PMC4915662; doi:10.1371/journal.pmed.1002041)
Supplement: S2 Text — Data analysis plan. (DOCX) [file pmed.1002041.s011.docx]

**Data analysis plan**

The data analysis plan was developed after a scientific workshop held on the 6^th^ February 2014 where it was decided to build and validate a prognostic instrument estimating the probability of full bladder recovery after spinal cord injury based on the EMSCI data. The final analytic strategy for the model derivation was defined after completing data checks by the two statistical advisors to this project, Dr. Alfons G. Kessels and Professor Lucas M. Bachmann on the 25^th^ August 2014. The final decision regarding the analytic strategy of model validation was made after completing the data checks of the validation cohort on the 20^th^ October 2014.
